# Mitigation of heat stress in wheat (*Triticum aestivum* L.) via regulation of physiological attributes using sodium nitroprusside and gibberellic acid

**DOI:** 10.1186/s12870-023-04321-9

**Published:** 2023-06-06

**Authors:** Xueping Zhang, Mingjun Ma, Chengcheng Wu, Shoucheng Huang, Subhan Danish

**Affiliations:** 1 College of Agriculture, Anhui Science and Technology University, Fengyang, 233100 China; 2 College of Life and Health Science, Anhui Science and Technology University, Fengyang, 233100 China; 3 Department of Soil Science, Faculty of Agricultural Sciences and Technology, Bahauddin Zakariya University, Multan, Punjab Pakistan

**Keywords:** Antioxidants, Chlorophyll, Growth attributes, Gibberellic acid, Heat stress, Sodium nitroprusside

## Abstract

Heat stress poses a threat to plants in arid and semiarid regions, leading to soil salinization and plant mortality. Researchers are exploring remedies to alleviate these effects, including using gibberellic acid (GA3) to regulate plant enzymes and antioxidants. Additionally, sodium nitroprusside (SNP) is gaining attention, but its combined effect with GA3 requires further research. To address this gap, we investigated the effects of GA3 and SNP on plants under heat stress conditions. For that, wheat plants were cultivated under 40 °C for 6 h per day (15 days). Sodium nitroprusside (donor of NO and SNP) and gibberellic acid (GA3), respectively, with 100 µM and 5 µg/ml concentrations, were applied as foliar sprays at 10 days after sowing (DAS). Results showed that SNP + GA3 treatment had the highest plant height (4.48% increase), plant fresh weight (29.7%), plant dry weight (87%), photosynthetic rate (39.76%) and stomatal conductance (38.10%), and Rubisco (54.2%) compared to the control. Our findings indicate a significant increase in NO, H_2_O_2_, TBARS, SOD, POD, APX, proline, GR, and GB that greatly scavenged reactive oxygen species (ROS) for decreasing the adverse effect of stress. Such findings confirmed the efficacy of the combined treatment of SNP + GA3 under high-temperature stress compared to the solitary application of GA3, SNP, and control. In conclusion, using SNP + GA3 is a better strategy for mitigating heat stress in wheat than individual applications. Further research is recommended to validate the effectiveness of SNP + GA3 in other cereal crops.

**Graphical Abstract:**

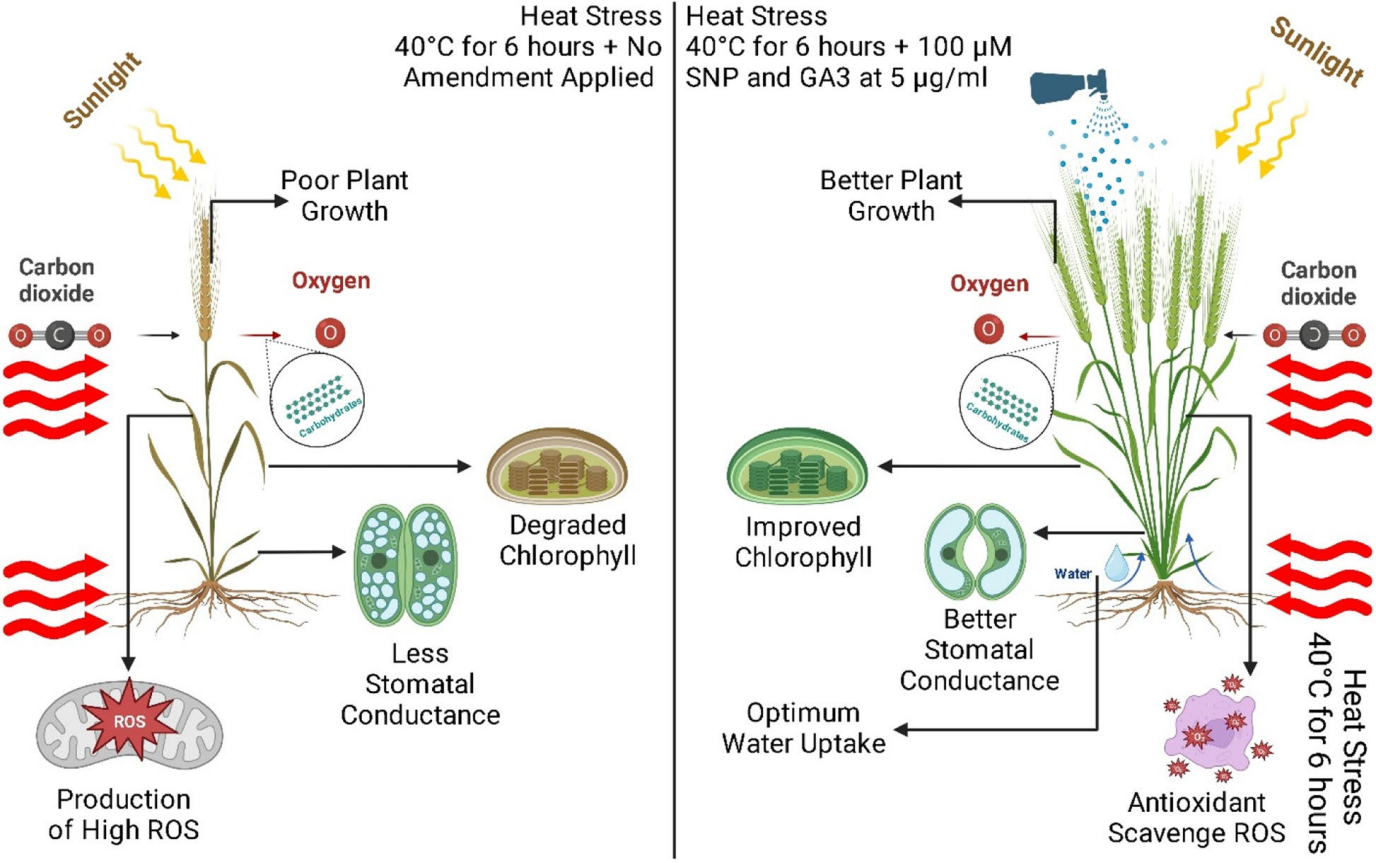

## Introduction

**Fig. 1:**
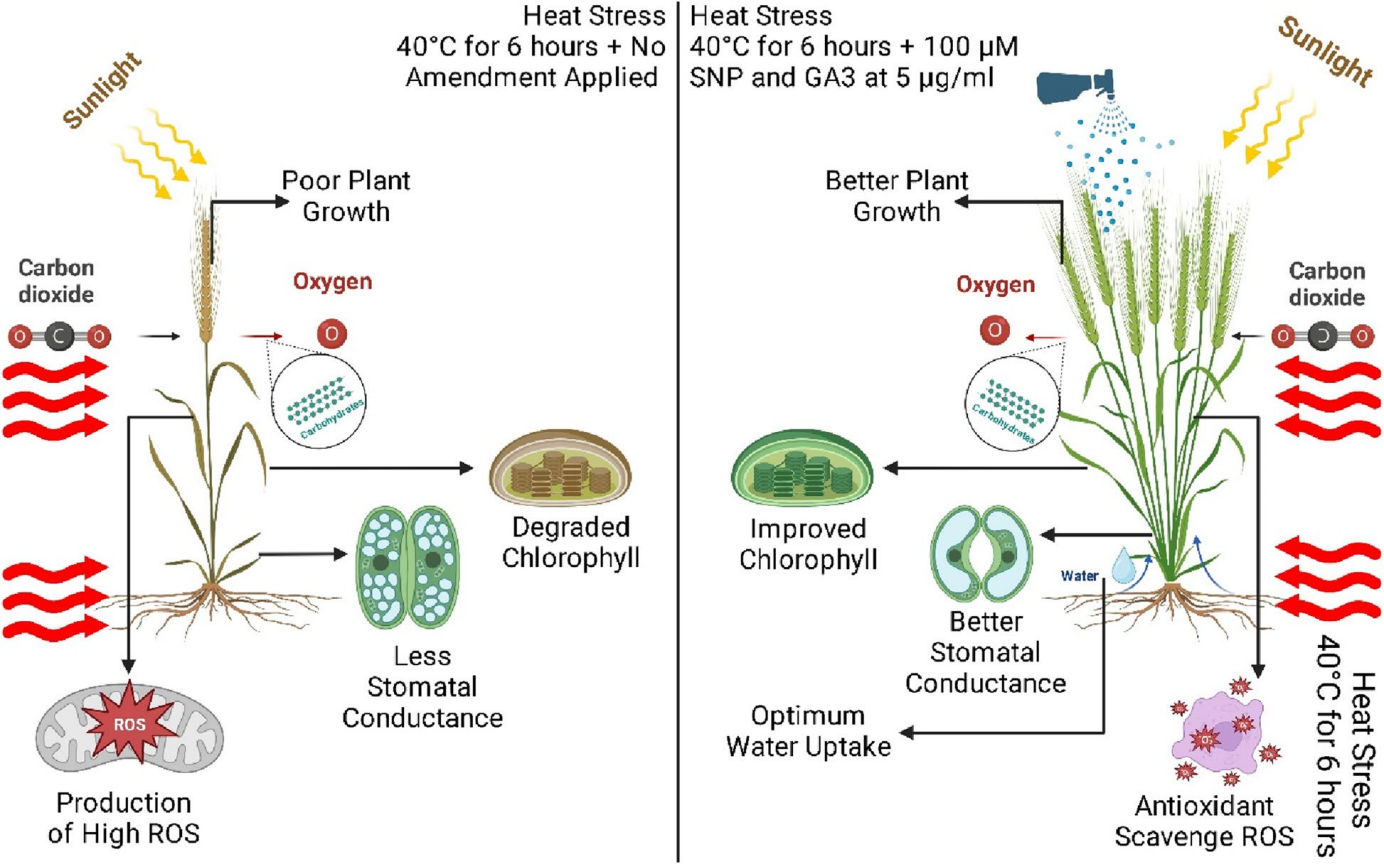
Graphical abstract

Heat stress can significantly impact plant growth and yield [1, 2]. When temperatures rise above a certain threshold, usually around 30 °C for most crops [3], plants experience physiological and metabolic changes that can negatively affect their growth and productivity [4] (Fig. 1). Several of the highest mutual belongings of heat stress on plants is a decline in photosynthesis. High temperatures can damage the photosynthetic apparatus, reduce chlorophyll content, and alter the balance between carbon assimilation and respiration [5]. Furthermore, heat stress has been identified to induce plants to experience an oxidative burst resulting in membrane lipid peroxidation, pigment bleaching, protein degradation, enzyme inactivation, and damage to macromolecules. [6], disrupt cell differentiation and elongation, degrade the cytoskeleton, and inhibit chloroplast activity [7]. The plant's susceptibility to heat stress is influenced by different stages of development and stress severity levels [8]. This can control a reduction in plant biomass and yield. Heat stress can also cause changes in plant morphology, such as a reduction in leaf size and root growth. This can affect the plant's ability to absorb water and nutrients from the soil, leading to drought stress and nutrient deficiencies [6]. Furthermore, Rubisco can become less efficient at high temperatures in fixing carbon dioxide for several reasons. Firstly, heat can cause the enzyme to denature or lose its three-dimensional structure, which is essential for its function [9]. This leads to a decrease in Rubisco's catalytic activity and can limit the rate of carbon assimilation by the plant. Secondly, heat stress can affect the balance of Rubisco's subunits, leading to an imbalance between the large and small subunits of the enzyme. This can result in the formation of non-functional Rubisco complexes, which further impairs the enzyme's efficiency in carbon fixation [9].

Sodium nitroprusside (SNP) has been shown to benefit plants under heat stress conditions [10]. SNP is a nitric oxide (NO) donor and NO has been shown to play a role in plant responses to environmental stresses, including heat stress [11]. Studies have shown that treating plants with SNP can alleviate some of the negative effects of heat stress, including reducing oxidative stress, improving photosynthesis, and increasing antioxidant activity [12]. SNP can also enhance the activity of enzymes involved in stress signaling and help maintain the integrity of the plant cell membrane [13].

On the other hand, gibberellic acid (GA3) is a plant hormone that has been shown to have a potential role in mitigating the negative effects of heat stress on plant growth and yield [14–16]. Studies have shown that treating plants with GA3 can increase the activity of enzymes involved in antioxidant defense mechanisms [17, 18], such as catalase and peroxidase, which can help reduce oxidative stress caused by heat [19]. GA3 has also been found to increase the accumulation of osmoprotectants such as proline and soluble sugars, which can help plants cope with heat stress-induced water deficit [20]. In addition, GA3 has been found to enhance the photosynthetic rate and carbon assimilation in plants under heat stress conditions [21]. This can help improve plant growth and yield under high-temperature conditions. However, the effectiveness of SNP and GA3 in mitigating heat stress in plants can depend on several factors, such as plant species, growth stage, concentration, and application method. Further research is needed to determine the optimal GA3 treatment regime for different crops under heat stress conditions.

Wheat is one of the world’s most important crops, providing a significant source of calories and protein for human consumption [22]. Climate change is expected to exacerbate heat stress in many wheat-growing regions, making it more difficult to achieve food security and meet the growing demand for wheat [23]. Heat stress is a major threat to wheat production in many regions, particularly areas already affected by water scarcity and other environmental stresses [24]. Research is needed to understand better the specific amendments that can help mitigate the impact of heat stress on wheat production. The present study aims to investigate the individual and combined effects of Gibberellic acid (GA3) and Sodium Nitroprusside (SNP) on wheat growth and yield under normal and heat stress conditions. By exploring the potential use of these two plant growth regulators for wheat under heat stress, this study aims to fill the existing knowledge gap in this area. It is hypothesized that the combined application of GA3 and SNP may be more effective in improving wheat growth and yield under heat stress than their applications.

## Material and methods

### Seeds sterilization

Wheat (Tr*iticum aestivum* L.), the seeds of cultivar Akbar 2019, purchased from the certified seed dealer of the Government of Punjab Pakistan, were subjected to surface sterilization using 5% sodium hypochlorite, three washes with ethanol (95%) and successive washing with double-distilled water [25].

### Incubation for germination

For the experiment, wheat seeds were incubated for germination at a diurnal/nocturnal warmth of 25/18 °C, using a 12-h photoperiod (PAR 300 µmol m^−2^ s^−1^), afterward comparative moistness of 65 ± 5%. The incubation conditions were carefully monitored and maintained throughout the experiment to ensure optimal conditions for seed germination. The seeds were placed in an incubator with temperature control to maintain the diurnal/nocturnal temperature of 25/18 °C. A timer provided a 12-h photoperiod with a PAR of 300 µmol m^−2^ s^−1^ during the light phase. Relative humidity was maintained using a humidifier and monitored using a hygrometer.

### Nutrition

A total of five plants were cultivated per pot, each receiving 150 mL of full-strength Hoagland’s nourishment mixture [26] was applied every alternate day. At 10 days after sowing (DAS), seedling emergence was observed; the plants were exposed to a temperature of 40 °C for 6 h per day for 15 days, while all other growth conditions remained constant., the plants were transferred to optimal temperature conditions (25 °C) and allowed to recover. The experimental growth period continued for 30 days, during which the plants were grown under the same conditions. The control plants were kept at a constant temperature of 25 °C for 30 days throughout the experiment.

### Sodium nitroprusside and gibberellic acid collection and characteristics

Sodium nitroprusside dihydrate (puriss. p.a., ACS reagent, reag. Ph. Eur., ≥ 99% Sigma-Aldrich; Batch Number: BCCK0585) of Very Dark Red and Red-Brown, Crystalline Chunk form was purchased from a certified dealer. Gibberellic acid (GA3, BioReagent, suitable for plant cell culture, ≥ 90% gibberellin A3 basiS; Batch Number: BCCJ9719) of White powder form, with clear colorless solubility was also acquired from the same dealer.

### Sodium nitroprusside and gibberellic acid application rate

The foliar spray application of 100 µM SNP and GA3 at 5 µg/ml 10 days after sowing (DAS) was tested. The SNP concentration was determined based on the findings of a previous study [27]. The treatments were established in a randomized blocked design with three replicates (*n* = 3). The spray volume of the chemicals was quantified at 25 mL, and plants were sampled for various measurements 35 days after sowing (DAS).

### Treatment plan

There were 4 treatments: control (no GA3 and no SNP), GA3, SNP, and GA3 + SNP. All the treatments were applied to wheat plants grown under no heat stress (NoHS) and heat stress (HS) (Table 1).

**Table 1:** Treatment plan

Sr. No	Treatment Plan	Abbreviations
1	No GA3 + No SNP + No Heat Stress	Control + NoHS
2	5 µg/ml GA3 + No Heat Stress	GA3 + NoHS
3	100 µM SNP + No Heat Stress	SNP + NoHS
4	GA3 + SNP + No Heat Stress	GA3 + SNP + NoHS
5	No GA3 + No SNP + Heat Stress	Control + HS
6	5 µg/ml GA3 + Heat Stress	GA3 + HS
7	100 µM SNP + Heat Stress	SNP + HS
8	GA3 + SNP + Heat Stress	GA3 + SNP + HS

### Purpose of development attributes

The plants were prepped for measurement by rinsing under running tap water to remove any sand adhering to them, followed by blotting dry with a soft paper towel to remove any excess moisture. Plant length was determined using a meter scale, while fresh weight and dry weight were assessed using an electronic balance and a hot air oven set at 65 °C for 72 h, respectively.

### Photosynthetic characteristics measurements

Measurements of gas exchange of each treatment’s third fully expanded leaf were conducted using a CID-340 infrared gas analyzer (Photosynthesis System, Bio-science, Washington, DC, USA). Chlorophyll content was quantified using a SPAD 502 DL PLUS chlorophyll meter (Konica Minolta, Japan). The activity of ribulose 1,5-bisphosphate carboxylase/oxygenase (Rubisco, EC 4.1.1.39) was evaluated spectrophotometrically, according to Usuda's method [28].

### Measurement of hydrogen peroxide and Thiobarbituric Acid Reactive Substances (TBARS) concentrations

The substance of H_2_O_2_ stayed verified by seeing the process of [29].

The TBARS assay was employed to quantify lipid peroxidation per the protocol described [30].

### Determination of NO generation

The nitrite content was quantified using the method outlined to determine its concentration [31] with slight modifications, as outlined in [32]. The absorbance of the reaction mixture was measured at 540 nm, and the NO concentration was quantified using a calibration curve generated with sodium nitrite as a reference.

### Evaluate of action of antioxidant enzymes

The 200 mg of fresh leaf tissue was homogenized in an ice-cold extraction buffer and centrifuged at 15,000 × g for 20 min at 4 °C. The resultant supernatant was used to assess enzyme activity, with protein content determined by the method of Bradford [33]. The activity of SOD was quantified by using bovine serum albumin as a standard method [34, 35]. The CAT activity was quantified by measuring the inhibition of the photochemical reduction of nitro blue tetrazolium (NBT) [36]. Ascorbate peroxidase (APX) activity was quantified by measuring the decrease in H_2_O_2_ concentration at 240 nm after a slight modification of the original method [37] method. In contrast, glutathione reductase (GR) activity was assessed using the [38]. Oxidation of NADPH at 340 nm, in the presence of GSH, is employed as a method.

### Determination of proline gratified

The amount of proline present in leaf tissue (300 mg) was determined via the procedure [39]. Centrifugation of homogenized leaf tissue at 11,500 × g for 12 min yielded a supernatant which was then combined with 2.0 mL acid ninhydrin and 2.0 mL glacial acetic acid in a test tube. The mixture was then incubated in a water bath at 100 °C for 1 h, then added 4.0 mL toluene and vigorously stirred for 20–30 s. Subsequently, the absorbance of the upper, reddish-pink phase was measured using L-proline as a reference at 520 nm.

### Determination of glycine betaine content

The quantification of GB in leaves was achieved by employing the [40] betaine-periodate complex formation technique. The specifics of the procedure are elucidated. Approximation of Entirety Solvable Sugar with Trehalose Content. The quantification of GB in leaves was achieved by employing the [41] betaine-periodate complex formation technique. The facts of the process are explained in [42].

### Statistical analysis

The study implemented a standard statistical procedure to conduct a statistical analysis. [43]. A one-way analysis of variance (ANOVA) was performed, and the results were visualized using OriginPro 2021 software to assess the differences in the data [44]. Results were expressed as mean ± SE (*n* = 3), and the Fisher’s LSD test was utilized to assess significance at a *p*-value of < 0.05.

## Results

**Fig. 2:**
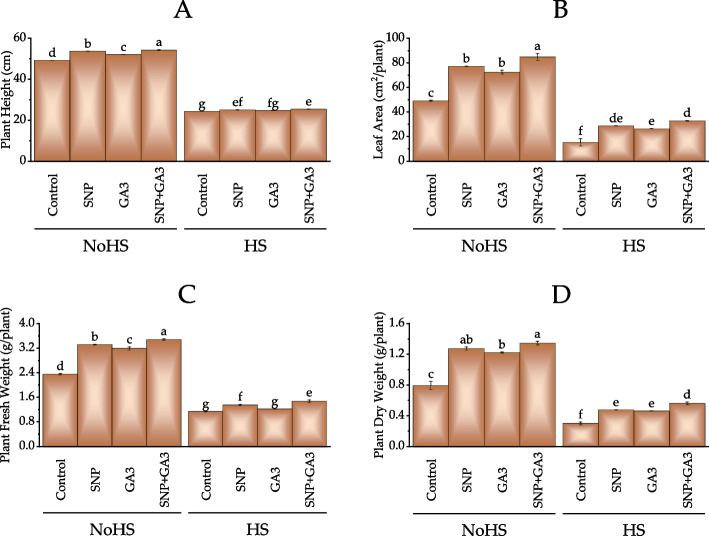
Effect of treatments on plant height (**A**), leaf area (**B**), plant fresh weight (**C**) and plant dry weight (**D**) of wheat under normal conditions and heat stress conditions. Bars values are average of 3 replicates. Different letters on bars showed significant change at *p* ≤ 0.05; Fisher LSD. NoHS = No Heat stress; HS = Heat stress

Under NoHS treatment, the control had a plant height of 49.15 cm. The SNP treatment significantly increased to 53.59 cm, representing a 9.02% increase compared to the control. The GA3 treatment also showed a significant increase in plant height to 51.96 cm, a 5.69% increase compared to the control. The SNP + GA3 treatment had the highest plant height at 54.11 cm, a 10.14% increase compared to the control. Under HS treatment, the control had a plant height of 24.28 cm. The SNP treatment slightly increased to 24.96 cm, a 2.84% increase compared to the control. The GA3 treatment showed a similar increase to 24.66 cm, representing a 1.63% increase compared to the control. The SNP + GA3 treatment had the highest plant height at 25.35 cm, a 4.48% increase compared to the control (Fig. 2A).

The leaf area of plants under NoHS conditions showed significant differences compared to the control group. The application of SNP, GA3, and SNP + GA3 resulted in an increase in leaf area by 57.17%, 47.3%, and 72.69%, respectively. Among all the treatments, the SNP + GA3 combination resulted in the most significant increase in leaf area, which was 84.63% compared to the control group. On the other hand, under HS conditions, the leaf area of plants in the control group showed a decrease of 68.77% compared to the control group under NoHS. However, applying SNP, GA3, and SNP + GA3 led to a significant increase in leaf area by 87.6%, 71.68%, and 114.38%, respectively. Among all the treatments, the SNP + GA3 combination showed the most significant increase in leaf area, which was 32.71% compared to the control group (Fig. 2B).

In NoHS treatment, the control had a plant fresh weight of 2.35 g/plant. The SNP treatment significantly increased to 3.31 g/plant, which is a 40.85% increase compared to the control. The GA3 treatment also showed a significant increase in plant fresh weight to 3.19 g/plant, representing a 35.44% increase compared to the control. The SNP + GA3 treatment had the highest plant fresh weight at 3.47 g/plant, a 47.64% increase compared to the control. For HS treatment, the control had a plant fresh weight of 1.13 g/plant. The SNP treatment slightly increased to 1.35 g/plant, a 19% increase compared to the control. The GA3 treatment showed a similar increase to 1.22 g/plant, representing a 7.4% increase compared to the control. The SNP + GA3 treatment had the highest plant fresh weight at 1.47 g/plant, a 29.7% increase compared to the control (Fig. 2C).

For NoHS conditions, all treatments resulted in an increase in plant dry weight compared to the control. The treatment with the highest dry weight was SNP + GA3, with a 69% increase over the control. The SNP and GA3 treatments also showed significant increases in dry weight compared to the control, with 61% and 54% increases, respectively. Under HS conditions, all treatments increased plant dry weight compared to the control, with the treatment SNP + GA3 showing the highest increase at 87%. The SNP and GA3 treatments also showed 58% and 54% increases, respectively, compared to the control. Overall, the results indicate that applying SNP and GA3, individually or in combination, can enhance plant growth and development under both normal and stress conditions (Fig. 2D).

**Fig. 3:**
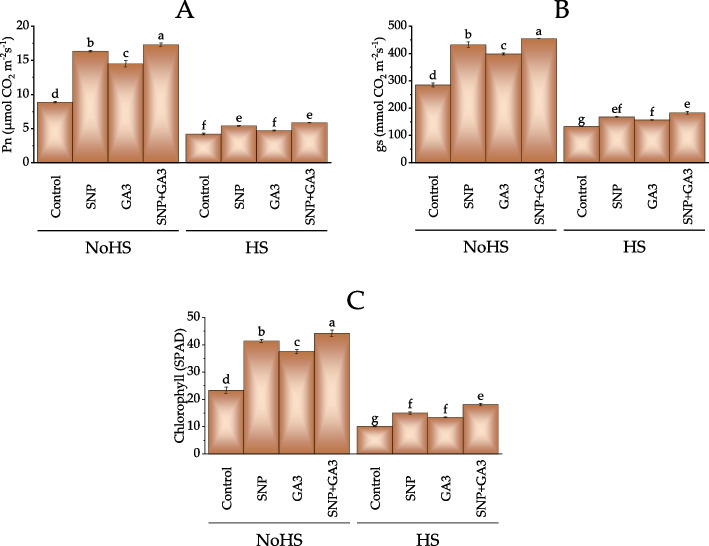
Effect of treatments on photosynthetic rate (Pn) (**A**), stomatal conductance (gs) (**B**) and chlorophyll (**C**) of wheat under normal conditions and heat stress conditions. Bars values are average of 3 replicates. Different letters on bars showed significant change at *p* ≤ 0.05; Fisher LSD. NoHS = No Heat stress; HS = Heat stress

In NoHS conditions, the photosynthetic rate was increased by 84.09% in the SNP + GA3 treatment group, the highest increase observed among all the treatment groups. The SNP treatment group showed a 84.61% increase in photosynthetic rate, followed by the GA3 treatment group with a 63.63% increase. In the case of HS conditions, the photosynthetic rate was increased by 39.76% in the SNP + GA3 treatment group, again the highest increase observed among all the treatment groups. The SNP treatment group showed a 28.84% increase in photosynthetic rate, followed by the GA3 treatment group with a 12.12% increase (Fig. 3A).

Under NoHS conditions, the stomatal conductance was significantly increased in plants treated with SNP (431.76, 52.29% increase) and SNP + GA3 (454.14, 59.64% increase) compared to the control (284.59). The GA3 treatment also increased stomatal conductance (397.96, 40.12% increase) compared to the control, although not as much as the SNP treatments. Under HS conditions, the stomatal conductance was also significantly increased in plants treated with SNP (167.44, 26.78% increase) and SNP + GA3 (182.47, 38.10% increase) compared to the control (132.18). The GA3 treatment also increased stomatal conductance (156.06, 18.13% increase) compared to the control, although not as much as the SNP treatments (Fig. 3B).

For NoHS conditions, the chlorophyll content of plants treated with SNP, GA3, and SNP + GA3 increased by 77.8%, 61.2%, and 89.9%, respectively, compared to the control. The plants treated with SNP + GA3 showed the highest increase in chlorophyll content, followed by SNP and GA3 treatments. At HS conditions, the chlorophyll content of plants treated with SNP, GA3, and SNP + GA3 increased by 47.9%, 32.1%, and 78.2%, respectively, compared to the control. Once again, the plants treated with SNP + GA3 showed the highest increase in chlorophyll content, followed by SNP and GA3 treatments (Fig. 3C).

**Fig. 4:**
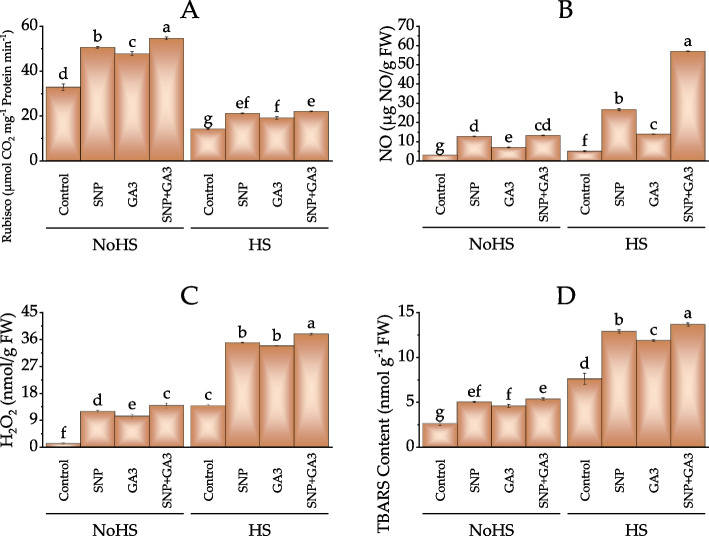
Effect of treatments on rubisco (**A**), NO (**B**), H_2_O_2_ (**C**) and TBARS content (**D**) of wheat under normal conditions and heat stress conditions. Bars values are average of 3 replicates. Different letters on bars showed significant change at *p* ≤ 0.05; Fisher LSD. NoHS = No Heat stress; HS = Heat stress

The Rubisco content of plants treated with different combinations of SNP and GA3 was investigated under NoHS and HS conditions. The results showed that under NoHS conditions, all three treatments, SNP, GA3, and SNP + GA3, increased the Rubisco content compared to the control. The plants treated with SNP + GA3 showed the highest increase in Rubisco content, with a 66.1% increase compared to the control. This was followed by the SNP treatment, which showed a 53.9% increase, and the GA3 treatment, which showed a 44.9% increase. Similarly, all three treatments increased the Rubisco content under HS conditions compared to the control. The plants treated with SNP + GA3 showed the highest increase in Rubisco content, with a 54.2% increase compared to the control. This was followed by the SNP treatment, which showed a 47.7% increase, and the GA3 treatment, which showed a 33.7% increase (Fig. 4A).

The results showed that the highest NO levels were observed in the samples treated with SNP + GA3, with a value of 56.91 µmol/g FW in HS conditions and 13.23 µmol/g FW in NoHS conditions. This represents an increase in NO levels of 49.6% and 332.7%, respectively, compared to the control treatment in the same conditions. The HS samples treated with SNP and GA3 also had higher NO levels than the control treatment, with values of 49.73 µmol/g FW and 45.19 µmol/g FW, respectively, respectively, representing increases of 30.5% and 18.7% compared to the control treatment. In NoHS conditions, the samples treated with SNP and GA3 had values of 12.78 µmol/g FW and 7.05 µmol/g FW, respectively, representing increases of 318.3% and 131.1% compared to the control treatment (Fig. 4B).

Under HS conditions, the H_2_O_2_ content of plants treated with SNP, GA3, and SNP + GA3 increased by 151.1%, 143.1%, and 171.7%, respectively, compared to the control. The plants treated with SNP + GA3 showed the highest increase in H_2_O_2_ content, followed by SNP and GA3 treatments. Under NoHS conditions, the H_2_O_2_ content of plants treated with SNP, GA3, and SNP + GA3 increased by 798.7%, 684.5%, and 947.8%, respectively, compared to the control. Once again, the plants treated with SNP + GA3 showed the highest increase in H_2_O_2_ content, followed by SNP and GA3 treatments (Fig. 4C).

The results showed that the highest level of TBARS was observed in the samples treated with SNP + GA3, with a value of 13.68 nmol/g protein in HS conditions and 5.39 nmol/g protein in NoHS conditions. This represents an increase in TBARS levels of 78.5% and 37.3%, respectively, compared to the control treatment in the same conditions. The HS samples treated with SNP and GA3 also had higher levels of TBARS compared to the control treatment, with values of 12.92 nmol/g protein and 11.89 nmol/g protein, respectively, representing increases of 68.8% and 55.5% compared to the control treatment. In NoHS conditions, the samples treated with SNP and GA3 had values of 5.04 nmol/g protein and 4.60 nmol/g protein, respectively, representing increases of 89.6% and 72.8% compared to the control treatment (Fig. 4D).

For HS condition, the combination treatment of SNP and GA3 showed the highest SOD activity with an average value of 19.50 U min^−1^ mg^−1^ FW protein, representing a 47.3% increase compared to the control group's average SOD activity of 13.25 U min^−1^ mg^−1^ FW protein. The GA3 treatment also significantly increased SOD activity, with an average value of 14.79 U min^−1^ mg^−1^ FW protein, representing an 11.5% increase compared to the control group. The SNP treatment showed a decrease in SOD activity, with an average value of 11.79 U min^−1^ mg^−1^ FW protein, representing a 10.9% decrease compared to the control group. At NoHS condition, all treatment groups showed lower SOD activity than the HS condition. The combination treatment of SNP and GA3 showed the highest SOD activity with an average value of 9.76 U min^−1^ mg^−1^ FW protein, representing a 50.7% decrease compared to the control group's average SOD activity of 7.04 U min^−1^ mg^−1^ FW protein. The SNP treatment showed a 24.6% decrease in SOD activity, with an average value of 9.34 U min^−1^ mg^−1^ FW protein, while the GA3 treatment resulted in a 16.1% decrease, with an average value of 7.91 U min^−1^ mg^−1^ FW protein.

Under the HS condition, the combination treatment of SNP and GA3 showed the highest CAT activity with an average value of 198.81 U min^−1^ mg^−1^ FW protein, representing a 45.1% increase compared to the control group's average CAT activity of 136.97 U min^−1^ mg^−1^ FW protein. The GA3 treatment also significantly increased CAT activity, with an average value of 169.83 U min^−1^ mg^−1^ FW protein, representing a 24.3% increase compared to the control group. The SNP treatment showed a moderate increase in CAT activity, with an average value of 183.34 U min^−1^ mg^−1^ FW protein, representing a 33.7% increase compared to the control group. At the NoHS condition, all treatment groups showed higher CAT activity compared to the NoHS condition. The combination treatment of SNP and GA3 showed the highest CAT activity with an average value of 79.12 U min^−1^ mg^−1^ FW protein, representing a 18.7% increase compared to the control group's average CAT activity of 66.62 U min^−1^ mg^−1^ FW protein. The SNP treatment showed a 12.8% increase in CAT activity, with an average value of 75.33 U min^−1^ mg^−1^ FW protein, while the GA3 treatment resulted in a 8.5% increase, with an average value of 72.44 U min^−1^ mg^−1^ FW protein.

At HS condition, the combination treatment of SNP and GA3 showed the highest APX activity with an average value of 7.86 U min^−1^ mg^−1^ FW protein, representing a 134.3% increase compared to the control group's average APX activity of 3.35 U min^−1^ mg^−1^ FW protein. The GA3 treatment also significantly increased APX activity, with an average value of 6.09 U min^−1^ mg^−1^ FW protein, representing an 81.4% increase compared to the control group. The SNP treatment showed a moderate increase in APX activity, with an average value of 7.21 U min^−1^ mg^−1^ FW protein, representing a 115.8% increase compared to the control group. For the NoHS condition, all treatment groups showed higher APX activity than the HS condition. The combination treatment of SNP and GA3 showed the highest APX activity with an average value of 2.89 U min^−1^ mg^−1^ FW protein, representing a 123.3% increase compared to the control group's average APX activity of 1.29 U min^−1^ mg^−1^ FW protein. The SNP treatment showed a 102.3% increase in APX activity, with an average value of 2.64 U min^−1^ mg^−1^ FW protein, while the GA3 treatment resulted in a 73.4% increase, with an average value of 2.24 U min^−1^ mg^−1^ FW protein (Table 2).

**Table 2:** Effect of treatments on SOD, CAT and APX of wheat under normal conditions and heat stress conditions

Stress	Treatment	SOD (U min^−1^ mg^−1^ FW Protein)	CAT (U min^−1^ mg^−1^ FW Protein)	APX (U min^−1^ mg^−1^ FW Protein)
HS	Control	13.25bc	136.97d	3.35d
	SNP	11.79c	183.34b	7.21b
	GA3	14.79b	169.83c	6.09c
	SNP + GA3	19.50a	198.81a	7.86a
NoHS	Control	7.04f	66.62f	1.29 g
	SNP	9.34de	75.33e	2.64ef
	GA3	7.91ef	72.44ef	2.24f
	SNP + GA3	9.76d	79.12e	2.89de

Values are average of 3 replicates. Different letters showed significant change at *p* ≤ 0.05; Fisher LSD. *NoHS* No Heat stress, *HS* Heat stress, *ABA* Abscisic acid, *SOD* Superoxide dismutase, *CAT* Catalase, *APX* Ascorbate peroxidase

In the case of HS condition, the combination treatment of SNP and GA3 showed the highest GR activity with an average value of 5.93 U min^−1^ mg^−1^ FW protein, representing a 72.4% increase compared to the control group's average GR activity of 3.44 U min^−1^ mg^−1^ FW protein. The SNP treatment also significantly increased GR activity, with an average value of 5.22 U min^−1^ mg^−1^ FW protein, representing a 51.7% increase compared to the control group. The GA3 treatment showed a moderate increase in GR activity, with an average value of 4.89 U min^−1^ mg^−1^ FW protein, representing a 41.7% increase compared to the control group. In HS condition, all treatment groups showed higher GR activity than the NoHS condition. The combination treatment of SNP and GA3 showed the highest GR activity with an average value of 2.32 U min^−1^ mg^−1^ FW protein, representing a 66.9% increase compared to the control group's average GR activity of 1.39 U min^−1^ mg^−1^ FW protein. The SNP treatment also resulted in a significant increase in GR activity, with an average value of 2.22 U min^−1^ mg^−1^ FW protein, representing a 59.0% increase compared to the control group. The GA3 treatment showed a moderate increase in GR activity, with an average value of 2.09 U min^−1^ mg^−1^ FW protein, representing a 51.2% increase compared to the control group.

Under HS treatment, the control had a Proline content of 17.68 µmol g^−1^ FW. The SNP treatment showed a slight increase to 18.86 µmol g^−1^ FW, representing a 6.67% increase compared to the control. The GA3 treatment had a Proline content of 18.53 µmol g^−1^ FW, which was 4.88% higher than the control. The SNP + GA3 treatment resulted in the highest Proline content of 19.29 µmol g^−1^ FW, indicating a significant increase of 9.09% compared to the control. Under NoHS treatment, the control had a significantly lower Proline content of 3.88 µmol g^−1^ FW. The SNP treatment had a Proline content of 7.23 µmol g^−1^ FW, representing an 87.63% increase compared to the control. The GA3 treatment showed the highest Proline content at 8.00 µmol g^−1^ FW, a significant increase of 106.19% compared to the control. The SNP + GA3 treatment had a Proline content of 9.09 µmol g^−1^ FW, indicating a 134.02% increase compared to the control.

Under HS treatment, the control had a GB content of 1.29 µmol g^−1^ DW. The SNP treatment significantly increased to 2.19 µmol g^−1^ DW, representing a 69.77% increase compared to the control. The GA3 treatment also showed a significant increase in GB content with 2.02 µmol g^−1^ DW, a 56.59% increase compared to the control. The SNP + GA3 treatment had a GB content of 2.32 µmol g^−1^ DW, indicating a significant increase of 79.07% compared to the control. For NoHS treatment, the control had a significantly lower GB content of 0.57 µmol g^−1^ DW. The SNP treatment had a GB content of 0.86 µmol g^−1^ DW, representing a 50.88% increase compared to the control. The GA3 treatment resulted in a higher GB content of 0.79 µmol g^−1^ DW, a 38.60% increase compared to the control. The SNP + GA3 treatment showed the highest GB content at 0.89 µmol g^−1^ DW, indicating a significant increase of 56.14% compared to the control. The results show that stress induction significantly affects GB content, and the response varies depending on the stress inducer and stress conditions. In HS treatment, all stress inducers resulted in a significant increase in GB content compared to the control, with the SNP + GA3 treatment showing the highest increase of 79.07%.

In HS treatment, the control had a TSS content of 54.16 mg g^−1^ DW. The SNP treatment significantly increased to 70.67 mg g^−1^ DW, representing a 30.40% increase compared to the control. The GA3 treatment also showed a significant increase in TSS content with 67.68 mg g^−1^ DW, a 24.76% increase compared to the control. The SNP + GA3 treatment had a TSS content of 74.58 mg g^−1^ DW, indicating a significant increase of 37.60% compared to the control. For NoHS treatment, the control had a significantly lower TSS content of 25.41 mg g^−1^ DW. The SNP treatment had a TSS content of 29.99 mg g^−1^ DW, representing a 18.06% increase compared to the control. The GA3 treatment resulted in a higher TSS content of 28.18 mg g^−1^ DW, a 10.93% increase compared to the control. The SNP + GA3 treatment showed the highest TSS content at 32.41 mg g^−1^ DW, indicating a significant increase of 27.60% compared to the control (Table 3).

**Table 3:** Effect of treatments on GR, proline, SB, and TSS of wheat under normal conditions and heat stress conditions

Stress	Treatment	GR (U min^−1^ mg^−1^ FW Protein)	Proline (µmol g^−1^ FW)	GB (µmol g^−1^ DW)	TSS (mg g^−1^ DW)
HS	Control	3.44d	17.68c	1.29d	54.16d
	SNP	5.22b	18.86ab	2.19b	70.67b
	GA3	4.89c	18.53b	2.02c	67.68c
	SNP + GA3	5.93a	19.29a	2.32a	74.58a
NoHS	Control	1.39 g	3.88 g	0.57 g	25.41 g
	SNP	2.22ef	8.00e	0.86e	29.99f
	GA3	2.09f	7.23f	0.79f	28.18f
	SNP + GA3	2.32e	9.09d	0.89e	32.41e

Values are average of 3 replicates. Different letters showed significant change at *p* ≤ 0.05; Fisher LSD. *NoHS* No Heat stress, *HS* Heat stress, *GR* Glutathione reductase, *GB* Glycine betaine, *TSS* Total soluble sugar

## Discussion

Heat stress can have direct and indirect effects on plant growth, ultimately leading to a reduction in plant yield. The direct effects of heat stress on plant growth include damage to cell membranes, reduced photosynthesis, and inhibition of enzyme activity [45]. Indirect effects include alterations in plant metabolism, water and nutrient uptake changes, and increased susceptibility to pests and diseases. One of the mechanisms by which heat stress can lead to plant damage is by increasing the production of reactive oxygen species (ROS), which are highly reactive molecules that can cause cellular damage [46]. ROS can be produced as a byproduct of normal plant metabolism, but heat stress can increase ROS production beyond the plant's ability to neutralize them, leading to oxidative stress [47]. Plants have a range of antioxidant systems in place to counteract the harmful effects of ROS. These include enzymes such as superoxide dismutase, catalase, peroxidase, and non-enzymatic antioxidants such as glutathione and ascorbate. When plants are exposed to heat stress, they often increase their production of these antioxidants as a defense mechanism [47]. However, while these antioxidant systems can help mitigate the effects of heat stress, they may not be sufficient to fully protect the plant from damage. Additionally, diverting resources towards antioxidant production may come at the expense of other processes such as growth and reproduction [47]. Similar results were also obtained in current study where heat stress caused a significant decline in wheat growth attributes, i.e., plant height, leaf area, plant fresh weight, plant dry weight (Fig. 1). It was also noted that heat stress caused significant decrease in photosynthetic rate and stomatal conductance. This decline was mainly associated with the minimization of chlorophyll in the wheat plants leaves (Fig. 2). Rubisco (Ribulose-1,5-bisphosphate carboxylase/oxygenase) is an enzyme that plays a key role in photosynthesis by fixing carbon dioxide from the atmosphere into organic compounds.

However, under high temperature conditions, Rubisco activity can decrease, reducing photosynthesis and plant growth. Our results are also in line with above argument. A significant decline in Rubisco under heat stress and improvement in SNP and GA3 validated the effectiveness of treatments. GA3 increases the amount of Rubisco protein in leaves via modulation of Rubisco activase activity. This improvement in Rubisco is directly associated with improvement in photosynthesis, which was vital in improving crop growth under stress conditions [48, 49]. On the other hand, SNP can reduce the oxygenase activity of Rubisco, allowing the enzyme to fix carbon dioxide and improve photosynthesis more effectively [50]. Similar kinds of improvements were also noted in the current study. TBARS (thiobarbituric acid reactive substances) measure oxidative stress and lipid peroxidation in cells. High temperatures can cause oxidative stress and lipid peroxidation in plant cells, leading to cell damage and death. Measuring TBARS levels can help to evaluate the extent of oxidative stress in plant cells under heat stress [51].

Sodium nitroprusside (SNP) is classified as a nitric oxide (NO) donor, which refers to its ability to release NO upon metabolism by the plant. Nitric oxide is a signaling molecule that participates in various physiological processes in plants, including responses to stress [52]. One potential mechanism by which SNP can protect plants from heat stress is modulating the plant's antioxidant systems [53]. Treatment with SNP can increase the activity of antioxidant enzymes such as superoxide dismutase, APX, and glutathine reductase (GR), which can attenuate the levels of reactive oxygen species (ROS) in the plant and thus prevent oxidative damage [53]. Moreover, SNP can elevate non-enzymatic antioxidants, such as ascorbate and glutathione, thereby augmenting the plant's capacity to counteract oxidative stress [54]. Additionally, SNP has been shown to modulate the expression of heat shock proteins (HSPs), a class of proteins that aid in safeguarding plants against heat stress by promoting the stabilization of cellular structures and averting protein damage. Studies have documented that SNP treatment can stimulate the expression of HSPs in plants, thereby providing further defense against heat stress [55]. Applying single nucleotide polymorphisms (SNPs) is a potentially effective means of reducing the harmful effects of heat stress on plant growth and development. Osmoprotectants, including proline, glycine betaine (GB), and trehalose, can act as signaling molecules to protect against enzyme denaturation, membrane stabilization, and protection of photosynthetic pigments due to their abilities to scavenge reactive oxygen species (ROS) and to help maintain osmotic homeostasis. [56]. The rise in such osmolytes in the present report indicates that NO and ABA treatments can enhance heat tolerance [57].

Treatment with GA3 has been demonstrated to increase the activity of superoxide dismutase and catalase. These two antioxidant enzymes may decrease the concentration of reactive oxygen species (ROS) and, thus, potentially reduce oxidative damage in plants [58]. Additionally, GA3 treatment can increase the levels of non-enzymatic antioxidants such as ascorbate and glutathione, further enhancing the plant's ability to counteract oxidative stress. Another way that GA3 can help alleviate heat stress is by promoting the synthesis of osmoprotectants [20]. Osmoprotectants are compounds that help maintain cell turgor and stability under water stress conditions and can also have protective effects against other types of stress, including heat stress [59]. Studies have shown that GA3 treatment can increase the synthesis of osmoprotectants such as proline and soluble sugars, which can help to protect plants from the negative effects of heat stress [60].

## Conclusion

It is concluded that 100 µM SNP and GA3 at 5 µg/ml can potentially minimize the adverse effects of heat stress on wheat. SNP and GA3 can improve wheat growth under heat stress compared to control. However, their combined application as an amendment imposed a synergetic impact which has better potential for wheat growth under heat stress enhancement. SNP + GA3 also efficiently regulates the antioxidants that play an important role in the regulation of stomatal conductance and improvement in chlorophyll contents. Growers should apply 100 µM SNP and GA3 at 5 µg/ml under heat stress on wheat. Further investigations are also suggested at the field level under different cereal crops to declare 100 µM SNP and GA3 at 5 µg/ml as the best application rate and amendment again heat stress.

## Data Availability

All the data is present inside the manuscript. There is no supplementary file.
